# Safety and immunogenicity of Biological E's typhoid conjugate vaccine TYPHIBEV^Ⓡ^ concomitantly administered with measles rubella vaccine: a phase IV prospective, multicenter study

**DOI:** 10.1016/j.ijregi.2025.100611

**Published:** 2025-02-25

**Authors:** Subhash Thuluva, Ramesh V Matur, SubbaReddy Gunneri, Rammohanreddy Mogulla, Chirag Dhar, Vijay Yerroju, Nagaganesh Balne, Bheemisetty S Chakravarthy, Manish Narang, Niranjana S Mahantshetti, Anil Kumar Pandey

**Affiliations:** 1 Biological E Limited, Hyderabad, India; 2 King George Hospital, Visakhapatnam, India; 3 GTB Hospital, Delhi, India; 4 KLES Dr. Prabhakar Kore Hospital & Medical Research Centre, J.N Medical College, Belagavi, India; 5 ESIC Medical College & Hospital, Faridabad, India

**Keywords:** Typhoid conjugate vaccine (TCV), Measles vaccine, Adverse events (AEs), Immune non-interference, National Immunization Program

## Abstract

**Objectives:**

Typhoid infections remain one of the major health concerns worldwide. Vaccines that prevent typhoid, particularly, the conjugated polysaccharide ones, are an important tool in addressing this major health concern. Here, we report on the safety profile of Biological E's TYPHIBEV^Ⓡ^ when administered to infants, children, and young adults and immunogenic non-interference data of TYPHIBEV when co-administered with the measles rubella (MR) vaccine to infants.

**Methods:**

This phase IV multicenter, open-label study enrolled 1252 healthy subjects aged 6 months to 45 years, stratified into three age subsets. In the 6 months to 2 years age group, 400 subjects aged 9-12 months were randomly allocated 1:1 to receive TYPHIBEV^Ⓡ^ and MR vaccine or MR vaccine only. Blood samples were collected at baseline, day 28 (anti-measles immunoglobulin G by enzyme-linked immunosorbent assay), and day 42 (anti-Vi immunoglobulin G by enzyme-linked immunosorbent assay). Solicited adverse events (AEs) were monitored for 7 days after vaccination, whereas unsolicited events, serious AEs, and medically attended events were recorded throughout.

**Results:**

TYPHIBEV^Ⓡ^ was well-tolerated, with injection site pain (5.5%), pyrexia (2.5%), and swelling (2%) being the most frequent AEs. One serious AE of pyrexia was unrelated to the vaccine. Seroprotection rates for Vi and measles antigens were comparable between groups.

**Conclusions:**

No safety signals were identified during this study and no immunogenic inference was seen when TYPHIBEV was co-administered with MR.

**Trial registration:**

CTRI/2022/02/040285

## Highlights

• Typhoid conjugate vaccines induce long-lasting T-cell responses, superior to Vi polysaccharide vaccines.
• TYPHIBEV is World Health Organization–prequalified after phase I-III trials and now in routine use.
• No safety signals when TYPHIBEV is co-administered with measles rubella in this phase IV trial.
• TYPHIBEV and measles component of measles rubella vaccine show no immunogenic interference.
• No safety signals with data in 1000+ participants aged 6 months to 45 years.

## Introduction

The gram-negative *Salmonella* Typhi bacteria cause typhoid fever, a serious systemic infection, which is one of the major health concerns worldwide. As per the US Centers for Disease Control and Prevention, an estimated 11-21 million cases of typhoid fever and 135,000-230,000 deaths occur every year [1]. Of these cases, about 10 million are estimated to occur in India every year [2]. Typhoid is transmitted mainly by the feco-oral route and occurs in areas where hygiene is compromised [3,4]. Although antibiotics continue to be used to treat typhoid, emergence of resistance to various antibiotic classes is adding complexity to the therapeutic approach [5]. Our dependency on antibiotic treatment is likely to be reduced by use of vaccines against *S.* Typhi [6]. Of particular importance is the development of new typhoid conjugate vaccines that use *S.* Typhi Vi polysaccharide antigen conjugated to a protein, thereby inducing T cell–dependent immune responses and immune memory [7]. Apart from the World Health Organization (WHO) and the Indian Academy of Pediatrics, many countries including Pakistan, Liberia, Zimbabwe, Malawi, Sierra Leone, Nepal, and Bangladesh have introduced or formally recommended typhoid conjugate vaccines for children as young as 6 months [8,9].

Biological E's (BE) typhoid Vi-CRM_197_ glyco-conjugate vaccine (TYPHIBEV^Ⓡ^ or BE-typhoid conjugate vaccine [TCV]) was developed by conjugating Vi polysaccharide with the CRM_197_ carrier protein. The Vi-CRM_197_ conjugate vaccine has been found to have an acceptable safety profile and has been found to be more immunogenic than a licensed Vi polysaccharide vaccine [10]. TYPHIBEV^Ⓡ^ has undergone a stringent clinical development process that led to its marketing authorization by the national regulatory agency of India. A phase I study was conducted in healthy adults aged 18-45 years with a single dose of TYPHIBEV^Ⓡ^ and an acceptable safety and tolerability profile was established. A phase II/III study conducted in healthy infants, children, and adults demonstrated that TYPHIBEV^Ⓡ^ was non-inferior to the licensed comparator vaccine in terms of immunogenicity, overall safety, and reactogenicity in participants aged 6 months to 45 years [11]. An additional phase III study was also conducted in infants aged 6 months to adults aged 45 years to expand the safety cohort (manuscript under review) of TYPHIBEV^Ⓡ^ .

An important question that needed to be addressed was TYPHIBEV^Ⓡ^'s safety, tolerability, and immunogenicity when co-administered with the measles-containing vaccine (usually administered at 9-12 months of age). The WHO in its 2018 position paper prioritized co-administration of TCV with other childhood vaccines [12]. In this manuscript, we present the results of a phase IV study evaluating the safety and immunogenicity of TYPHIBEV^Ⓡ^ when co-administered with a licensed measles-rubella (MR) vaccine.

## Methods

### Study design and study population

This was a phase IV multicenter safety and immunogenicity study to assess the immune inference of TYPHIBEV^Ⓡ^ on measles immune response when co-administered with measles-containing vaccine in infants aged 9-12 months. In addition, the overall safety and tolerability of TYPHIBEV^Ⓡ^ was also evaluated in healthy subjects aged 6 months to 45 years. In total, 1252 participants were enrolled in this study from 11 sites across India (supplementary information) in accordance with the ethical principles defined in the Declaration of Helsinki, International Council for Harmonization Good Clinical Practices guidelines, and applicable local regulatory requirements. The ethics committee or institutional review board at each study site approved the protocol. Written informed consent was provided by all participants and/or their parents before enrollment into the study. The total duration of the study was 42 days.

Healthy participants of either gender aged ≥6 months to ≤45 years were included in the study. Anyone with a history of typhoid infection, vaccination against typhoid, any serious or chronic disease, use of immunosuppressants, allergic reaction to vaccine-related components, body temperature ≥38.0^°^C within 3 days before the day of vaccination, or unwillingness or inability to understand and follow the study procedures was excluded from the study recruitment. After screening, the participants who met all the inclusion and exclusion criteria were enrolled into the study.

Participants were enrolled and stratified in to three age subsets: infants and toddlers aged ≥6 months to <2 years (n = 532), children and adolescents aged ≥2 years to <18 years (n = 360), and adults aged ≥18 years to ≤45 years (n = 360) were part of age subsets 1, 2, and 3, respectively. In addition, study subjects in age subset 1 aged between 9 and 12 months were randomly allocated in a 1:1 ratio to receive either co-administered TYPHIBEV^Ⓡ^ and MR vaccine (n = 200) or MR vaccine only (n = 200). In age subset 1, an additional 132 subjects (aged 6 months to <2 years) received only TYPHIBEV^Ⓡ^. All other participants in age subsubset 2 (aged 2 years to <18 years, n = 360) and age subset 3 (aged 18 years to 45 years, n = 360) received only TYPHIBEV^Ⓡ^.

### Procedure

A single dose of BE's typhoid Vi-CRM_197_ polysaccharide conjugate vaccine (TYPHIBEV^Ⓡ^) was administered intramuscularly in the deltoid muscle of upper arm for children aged 2 years and older, adolescents, and adults. For infants and toddlers aged ≥6 months to <2 years, the vaccine was administered intramuscularly in the vastus lateralis muscle on the anterolateral aspect of thigh.

Each dose of this monovalent TCV (0.5 ml of a single human dose in a vial) contains 25 µg of typhoid Vi polysaccharide conjugated to 16.7-100.0 µg of CRM_197_. A total of 5 µg of 2-phenoxyethanol was used as a preservative. In the sub-groups receiving MR, 0.5 ml of BE's MR vaccine was administered by subcutaneous injection in the antero-lateral aspect of the upper thigh. Each 0.5 ml dose of BE's MR vaccine contains the CAM-70 strain of the measles virus propagated in chicken embryo fibroblast cells (≥1000 cell culture infectious dose 50%) and the Wistar RA 27/3 strain of the Rubella virus propagated in MRC 5 cells (≥1000 cell culture infectious dose 50%).

For infants randomly allocated to receive TYPHIBEV^Ⓡ^ and MR, three visits were scheduled, with day 0 for screening and enrollment, one on day 28 to obtain blood samples for immunogenicity assessments and adverse event (AE) reporting, and a final visit on day 42. For those participants receiving only TYPHIBEV^Ⓡ^, only day 0 and 42 visits were scheduled, whereas infants randomly allocated to MR alone had two visits on day 0 and 28. All visits after enrollment allowed an additional 7-day window period to promote adherence. The study duration for those that received only MR was 35 days (28 + 7 days) and was 49 days (42 + 7 days) for all other participants. During the conduct of these studies, there were no major protocol deviations reported at any of the study sites. Few deviations pertaining to visits out of window period were notified to the ethics committees of the respective study sites.

### Randomization and blinding

For the age 9-12 months cohort, participants were randomly allocated 1:1 to receive either TYPHIBEV^Ⓡ^ and MR vaccine or MR vaccine only. Randomization was performed using PROC PLAN in SAS, version 9.4. The purpose of randomization was to eliminate treatment selection bias and ensure balance of prognostic factors across treatment groups. The random allotment sequence (randomization code) was concealed to reduce bias within the investigational group by Interactive Web Response System. This was an open-label study. However, the person in charge of the laboratory testing remained blinded to the treatment, and codes were used to link the subject and study (without any link to the treatment attributed to the subject) to each sample.

### Outcomes

The primary objective of this study was to evaluate the safety and tolerability of TYPHIBEV^Ⓡ^ in healthy subjects aged 6 months to 45 years. The secondary objective was to assess the potential interference between TYPHIBEV^Ⓡ^ and the MR vaccine when co-administered to infants aged 9-12 months.

Immunogenicity outcomes included anti-measles immunoglobulin (Ig)G assessment in those who received only MR or TYPHIBEV^Ⓡ^ and MR and anti-Vi IgG antibody assessment in those who received only TYPHIBEV^Ⓡ^ or TYPHIBEV^Ⓡ^ and MR. Geometric mean concentrations (GMC) at baseline (day 0) and day 28 (anti-measles IgG) or day 42 (anti-Vi IgG), and proportion of subjects seroprotected against measles at day 28 and against Vi antigen at day 42 were assessed. In addition, the proportion of participants achieving ≥4-fold increase in anti-measles and anti-Vi IgG antibody concentrations and their geometric mean fold increase from baseline were also evaluated. Seroprotection was defined as participants achieving ≥2.0 μg/ml for anti-Vi specific antibodies and ≥120 mIU/ml for anti-measles antibodies after vaccination.

The safety outcomes were the number and percentage of participants with solicited local and systemic adverse reactions and the number and percentage of subjects with unsolicited AEs, serious AEs, and medically attended AEs.

### Safety assessments

Each vaccinated study participant was observed for at least 30 minutes after vaccination for evidence of immediate reactogenicity. Each participant or child's parent/Legally Authorized Representative was instructed to complete a diary card for 7 consecutive days (day 0 to day 6) after the single dose of vaccination to record any solicited local or systemic reactions. Unsolicited AEs, serious AEs (SAEs) and medically attentive AEs were collected during the entire 42-day follow-up period after vaccination.

All AEs that occurred within the study period, regardless of severity and causality, were monitored and medically managed by the investigator until resolution. The severity of all AEs was graded as per the common terminology criteria for AEs (CTCAE V5.0) or division of AIDS table version 2.0. All AEs were assessed for relatedness to the study vaccine by the investigators.

For adults, all vital signs such as oral body temperature in Fahrenheit), pulse rate per minute, blood pressure (millimeters of mercury), and respiratory rate per minute were recorded. For children and adolescents, only oral/axillary body temperature was captured.

### Immunogenicity assays and assessments

Immunogenicity assays and assessments were conducted as part of the secondary outcomes in age subset 1. Briefly, anti-measles IgG and anti-Vi IgG serum antibodies were measured by enzyme-linked immunosorbent assay at approximately day 28 for measles and at day 42 for typhoid after the completion of single-dose immunization. A total of approximately 3.5-5.0 ml of whole blood sample was collected to assess the measles and typhoid immunogenicity at baseline (day 0) and again at days 28 and/or 42 after a single dose for the subjects in age subset 1. The measles IgG titer estimation for all serum samples was determined by the Siemens Enzygnost anti-measles IgG test (Siemens, Marburg, Germany). Anti-Vi IgG antibody titers were estimated by VaccZyme enzyme-linked immunosorbent assay kit manufactured by Binding Site. Any individual with an anti-measles IgG level ≥120 mIU/ml was considered to be seroprotected against measles and anyone with an anti-Vi IgG antibody concentration of ≥2.0 μg/mL considered to be seroprotected against typhoid.

### Statistical analyses

The demographic and baseline characteristics of study participants were presented in the intent-to-treat population, defined as all participants enrolled into the study. Data were analyzed by summary statistics. For primary safety analysis, safety population was used, defined as participants who entered the study and received the study vaccination. For continuous variables n, mean, SD, median, and range (minimum, maximum) were presented. For categorical data, frequencies and relative frequencies were computed.

Systemic and local tolerability, recorded in subject diaries, was summarized in a frequency table with percentages based on the number of observed values. Systemic tolerability was assessed through recording of body temperature and clinical symptomatology. The number and percentage of subjects with AEs, treatment emergent AEs, serious AEs, and cumulative incidence rates were presented overall by body system and preferred term. All reported AEs during the entire study period were summarized by calculating frequencies and relative frequencies and were listed including severity, relationship to the vaccine (causality), and action taken.

SAEs and medically attended AEs reported during the study were listed and analyzed for expectedness and causality. Only treatment emergent AEs, i.e. those events which were not present at baseline but started after the first vaccination or worsened with respect to severity after the start of treatment, were tabulated. Changes in body temperature from study start to end of study were analyzed descriptively and were part of study evaluation only in case of clinically significant changes.

The two-sided exact 95% confidence intervals were calculated for all the occurrence rates of reported AEs and SAEs during the study. Medical history, all AEs, and inter-current illnesses were coded using MedDRA, and concomitant medications were coded using the WHO drug dictionary.

For the *a priori* determined secondary end points of this study, anti-measles IgG and anti-Vi IgG antibody levels and their GMC were measured at baseline (day 0) and after the vaccination(s) (day 28 for anti-measles IgG antibodies and day 42 for anti-Vi IgG antibodies). In addition, the proportion of subjects seroprotected against measles antigen at day 28 and against Vi antigen at day 42 and the proportion of subjects achieving ≥4-fold increase in anti-measles and anti-Vi IgG antibody concentrations at day 28 from baseline were also measured. Geometric mean fold increases were also calculated. These measures were descriptively analyzed for those infants and toddlers that were in the subgroup that received BE-TCV only, MR only, or BE-TCV and MR.

A sample size of 200 each for the BE-TCV and MR arm and MR only arm was determined to for descriptive comparison of immune responses in the two arms. The total sample size of this phase IV study and another phase III safety-only study of BE-TCV were calculated together to exceed the minimum exposure of 3000 participants needed to identify any rare AEs (1 in 1000 AEs).

## Results

### Participants

**Figure 1:**
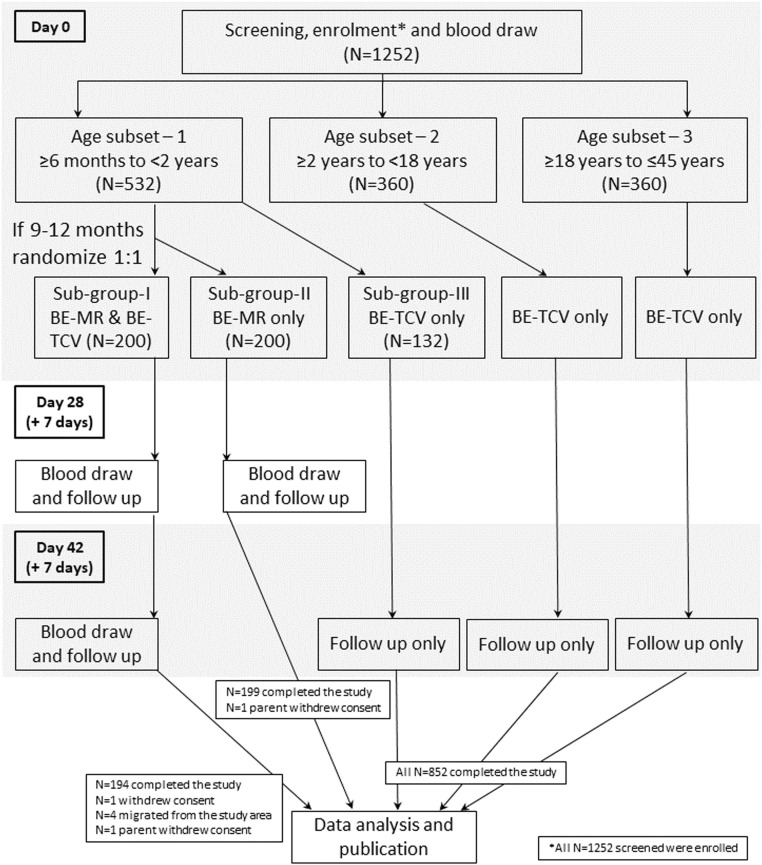
Study schema. MR, measles rubella; TCV, typhoid conjugate vaccine.

A total of 1252 participants were included in this phase IV study conducted between May 2022 and August 2022. All participants received the study vaccine, and safety analyses were performed in all these enrolled participants. A summary of the demographics of this intention-to-treat population is presented in Table 1. The mean age of those in age subset 1 randomly allocated to receive TYPHIBEV and MR or MR only were about the same at about 10 months. The remaining infants who received TYPHIBEV only had a mean age of about 10.5 months. The mean age was 8.9 ± 4.09 years in age subset II and 29.3 ± 7.24 years in age subset III. The gender distribution was roughly equal, with 49.1% being female. A study flow chart is depicted in Figure 1. Medical history, baseline disease characteristics, concomitant medications, and vaccination history of the study subjects are presented in Supplementary Tables 1-3.

**Table 1:** Clinical characteristics of participants.

Parameter/category	≥6 months to <2 years			≥2 years to <18 years	≥18 years to ≤45 years	Overall (N = 1252)
	TYPHIBEV^Ⓡ^ & MR (N = 200)	MR only (N = 200)	TYPHIBEV^Ⓡ^ (N = 132)	TYPHIBEV^Ⓡ^ (N = 360)	TYPHIBEV^Ⓡ^ (N = 360)	
Age	(months)	(months)	(months)	(years)	(years)	(years)
Mean	9.96	9.98	10.51	8.905	29.252	11.033
SD	0.79	0.82	4.02	4.0952	7.2352	12.9269
Median	9.92	9.87	9.22	9.040	29.010	5.555
Gender, N1 (%)						
Male	108 (54.0)	98 (49.0)	87 (65.9)	160 (44.4)	184 (51.1)	637 (50.9)
Female	92 (46.0)	102 (51.0)	45 (34.1)	200 (55.6)	176 (48.9)	615 (49.1)
Nationality						
Indian	200 (100)	200 (100)	132 (100)	360 (100)	360 (100)	1252 (100)
Height (cm)						
N1	200	200	132	360	360	1252
Mean	71.16	71.75	72.33	126.52	162.70	113.62
SD	4.454	4.573	6.019	20.450	7.730	40.456
Median	70.60	71.00	70.95	128.00	163.00	114.00
Range (min-max)	(62.0, 90.2)	(62.0, 90.0)	(65.0, 92.0)	(85.0, 171.0)	(145.0, 180.0)	(62.0, 180.0)
Weight (kg)						
N1	200	200	132	360	360	1252
Mean	8.32	8.41	7.98	27.57	61.23	29.05
SD	1.104	1.061	1.116	12.045	7.371	23.236
Median	8.50	8.60	8.00	25.55	60.75	19.00
Range (min-max)	(5.8, 10.8)	(6.0, 11.5)	(6.0, 12.4)	(10.0, 60.0)	(45.0, 85.0)	(5.8, 85.0)

MR, measles rubella; N_1_, subject count; N, sample size.

Note: The age were calculated using following formula: age (at vaccination) = round of ([vaccination date – birth date] ÷ 365.25) for years and round of ([vaccination date – birth date] ÷ 365.25) x 30.4375 for months.

Percentages were calculated using the column header group count as the denominator.

### Safety findings

A total of 200 study subjects received only an MR vaccine and the AE rates were 17.5% (35 of 200) in this subgroup. Of all the remaining study participants that received the TYPHIBEV^Ⓡ^ vaccine, 139 of 1052 (13.21%) reported at least one AE. A large majority of AEs were local and solicited in nature (∼10% of study subjects and ∼72% of all AEs) and reported in the 7-day post-vaccination period (∼12% of study subjects and ∼91% of all AEs). There was only one participant with an AE in the first 30 minutes after vaccination. Unsolicited AEs were reported in 12 participants at a frequency of 1.14%. There was one SAE reported in a female infant aged 9 months who received TYPHIBEV^Ⓡ^ and MR vaccine. Approximately 34 days after the vaccinations, the infant developed fever, loose stools, and vomiting for which she was hospitalized and discharged in a stable condition after about 3 days. This event was considered an SAE because of the hospitalization but, as per the principal investigator's opinion, was moderate in severity and not related to the study vaccine.

In total, there were eight medically attended AEs (0.8%) among those who received the TYPHIBEV^Ⓡ^ vaccine. These included cases of gastrointestinal disorders such as diarrhea and vomiting, pyrexia, gastroenteritis, cough/rhinorrhea, and a rash. When analyzed according to the system organ class classification of AEs, injection site pain, followed by pyrexia, were the most frequent AEs. An overview of the reported AEs, including solicited and unsolicited AEs, are presented in Table 2, and a summary of overall AEs by System Organ Class and Preferred Terms are listed in Table 3.

**Table 2:** Overview of subjects with AEs after vaccination - safety population (N = 1252) in the phase IV study.

Overview of AEs n (%) (95% CI) E	Age subset I			Age subset II TYPHIBEV^Ⓡ^ (N = 360)	Age subset III TYPHIBEV^Ⓡ^ (N = 360)
	Subgroup I MR + TYPHIBEV^Ⓡ^ (N = 200)	Subgroup II MR (N = 200)	Subgroup III TYPHIBEV^Ⓡ^ (N = 132)		
Overall AEs	22 (11.0) [7.02, 16.18] 32	35 (17.5) [12.50, 23.49] 41	15 (11.4) [6.50, 18.05] 16	49 (13.6) [10.24, 17.59] 60	53 (14.7) [11.23, 18.81] 63
AEs within 30 minutes post-vaccination	0 (0.0)	0 (0.0)	1 (0.8) [0.02, 4.15] 2	0 (0.0)	0 (0.0)
AEs within 7 days post-vaccination	21 (10.5) [6.62, 15.60] 27	35 (17.5) [12.50, 23.49] 41	15 (11.4) [6.50, 18.05] 16	47 (13.1) [9.75, 16.98] 58	46 (12.8) [9.51, 16.67] 54
Solicited local adverse event	16 (8.0) [4.64, 12.67] 16	25 (12.5) [8.26, 17.90] 27	10 (7.6) [3.69, 13.49] 11	39 (10.8) [7.82, 14.51] 50	40 (11.1) [8.06, 14.82] 46
Solicited systemic adverse event	8 (4.0) [1.74, 7.73] 11	10 (5.0) [2.42, 9.00] 14	5 (3.8) [1.24, 8.62] 5	8 (2.2) [0.96, 4.33] 8	7 (1.9) [0.79, 3.97] 8
Unsolicited adverse event	3 (1.5) [0.31, 4.32] 5	0 (0.0)	0 (0.0)	2 (0.6) [0.07, 1.99] 2	7 (1.9) [0.79, 3.97] 9
Serious adverse events	1 (0.5) [0.01, 2.75] 1	0 (0.0)	0 (0.0)	0 (0.0)	0 (0.0)
Adverse events leading to death	0 (0.0)	0 (0.0)	0 (0.0)	0 (0.0)	0 (0.0)
Medically attended adverse events	4 (2.0) [0.55, 5.04] 9	3 (1.5) [0.31, 4.32] 6	0 (0.0)	0 (0.0)	4 (1.1) [0.30, 2.82] 6
Discontinued due to adverse event	0 (0.0)	0 (0.0)	0 (0.0)	0 (0.0)	0 (0.0)

AE, adverse event; E, number of events; MR, measles rubella; N, the total number of subjects in each treatment group under the safety population.; n, number of subjects reported AEs.

% = Percentage of subjects; 95% CI = 95% confidence interval for the proportion of subjects.

The 95% CI estimated using the Clopper-Pearson exact method.

Subjects are counted only once within each preferred term and system organ class.

Percentages are based on the age subsets and treatments of Subjects in the specified treatment group under safety population.

AEs were coded using the Medical Dictionary of Regulatory Activities (MedDRA version 24.1).

**Table 3:** Summary of subjects with overall AEs by SOC and PT - safety population (N = 1252) in the phase IV study.

System organ class preferred term	Age subset I			Age subset II TYPHIBEV^Ⓡ^ (N = 360) n (%) (95% CI) E	Age subset III TYPHIBEV^Ⓡ^ (N = 360) n (%) (95% CI) E
	Subgroup I MR + TYPHIBEV^Ⓡ^ (N = 200) n (%) (95% CI) E	Subgroup II MR (N = 200) n (%) (95% CI) E	Subgroup III TYPHIBEV^Ⓡ^ (N = 132) n (%) (95% CI) E		
Number of Subjects with at least one AE	22 (11.0) [7.02, 16.18] 32	35 (17.5) [12.50, 23.49] 41	15 (11.4) [6.50, 18.05] 16	49 (13.6) [10.24, 17.59] 60	53 (14.7) [11.23, 18.81] 63
Eye disorders	0 (0.0)	0 (0.0)	0 (0.0)	1 (0.3) [0.01, 1.54] 1	0 (0.0)
Eye pruritus	0 (0.0)	0 (0.0)	0 (0.0)	1 (0.3) [0.01, 1.54] 1	0 (0.0)
Gastrointestinal disorders	3 (1.5) [0.31, 4.32] 5	1 (0.5) [0.01, 2.75] 1	0 (0.0)	0 (0.0)	1 (0.3) [0.01, 1.54] 1
Diarrhea	3 (1.5) [0.31, 4.32] 4	1 (0.5) [0.01, 2.75] 1	0 (0.0)	0 (0.0)	1 (0.3) [0.01, 1.54] 1
Vomiting	1 (0.5) [0.01, 2.75] 1	0 (0.0)	0 (0.0)	0 (0.0)	0 (0.0)
General disorders and administration site conditions	20 (10.0) [6.22, 15.02] 23	34 (17.0) [12.07, 22.94] 36	15 (11.4) [6.50, 18.05] 16	43 (11.9) [8.78, 15.75] 54	45 (12.5) [9.27, 16.37] 52
Chills	0 (0.0)	0 (0.0)	0 (0.0)	0 (0.0)	1 (0.3) [0.01, 1.54] 1
Fatigue	0 (0.0)	0 (0.0)	0 (0.0)	0 (0.0)	1 (0.3) [0.01, 1.54] 1
Injection site Erythema	1 (0.5) [0.01, 2.75] 1	6 (3.0) [1.11, 6.42] 6	2 (1.5) [0.18, 5.37] 2	10 (2.8) [1.34, 5.05] 10	5 (1.4) [0.45, 3.21] 5
Injection site Induration	0 (0.0)	0 (0.0)	0 (0.0)	1 (0.3) [0.01, 1.54] 1	5 (1.4) [0.45, 3.21] 5
Injection site pain	11 (5.5) [2.78, 9.63] 11	15 (7.5) [4.26, 12.07] 15	6 (4.5) [1.69, 9.63] 6	31 (8.6) [5.93, 12.00] 36	34 (9.4) [6.63, 12.95] 35
Injection site pruritus	0 (0.0)	1 (0.5) [0.01, 2.75] 1	0 (0.0)	0 (0.0)	0 (0.0)
Injection site rash	1 (0.5) [0.01, 2.75] 1	0 (0.0)	0 (0.0)	0 (0.0)	0 (0.0)
Injection site Swelling	4 (2.0) [0.55, 5.04] 4	5 (2.5) [0.82, 5.74] 5	3 (2.3) [0.47, 6.50] 3	3 (0.8) [0.17, 2.42] 3	1 (0.3) [0.01, 1.54] 1
Irritability Post-vaccinal	1 (0.5) [0.01, 2.75] 1	2 (1.0) [0.12, 3.57] 2	0 (0.0)	0 (0.0)	0 (0.0)
Pyrexia	5 (2.5) [0.82, 5.74] 5	7 (3.5) [1.42, 7.08] 7	5 (3.8) [1.24, 8.62] 5	4 (1.1) [0.30, 2.82] 4	4 (1.1) [0.30, 2.82] 4
Infections and infestations	1 (0.5) [0.01, 2.75] 1	0 (0.0)	0 (0.0)	0 (0.0)	0 (0.0)
Gastroenteritis	1 (0.5) [0.01, 2.75] 1	0 (0.0)	0 (0.0)	0 (0.0)	0 (0.0)
Musculoskeletal and connective tissue disorders	0 (0.0)	0 (0.0)	0 (0.0)	0 (0.0)	1 (0.3) [0.01, 1.54] 1
Pain in extremity	0 (0.0)	0 (0.0)	0 (0.0)	0 (0.0)	1 (0.3) [0.01, 1.54] 1
Nervous system disorders	0 (0.0)	0 (0.0)	0 (0.0)	5 (1.4) [0.45, 3.21] 5	3 (0.8) [0.17, 2.42] 3
Headache	0 (0.0)	0 (0.0)	0 (0.0)	5 (1.4) [0.45, 3.21] 5	3 (0.8) [0.17, 2.42] 3
Respiratory, thoracic, and mediastinal disorders	1 (0.5) [0.01, 2.75] 2	2 (1.0) [0.12, 3.57] 3	0 (0.0)	0 (0.0)	1 (0.3) [0.01, 1.54] 1
Cough	1 (0.5) [0.01, 2.75] 1	2 (1.0) [0.12, 3.57] 2	0 (0.0)	0 (0.0)	1 (0.3) [0.01, 1.54] 1
Rhinorrhea	1 (0.5) [0.01, 2.75] 1	1 (0.5) [0.01, 2.75] 1	0 (0.0)	0 (0.0)	0 (0.0)
Skin and subcutaneous tissue disorders	1 (0.5) [0.01, 2.75] 1	1 (0.5) [0.01, 2.75] 1	0 (0.0)	0 (0.0)	5 (1.4) [0.45, 3.21] 5
Pruritus	0 (0.0)	0 (0.0)	0 (0.0)	0 (0.0)	4 (1.1) [0.30, 2.82] 4
Rash	1 (0.5) [0.01, 2.75] 1	1 (0.5) [0.01, 2.75] 1	0 (0.0)	0 (0.0)	1 (0.3) [0.01, 1.54] 1

AE, adverse event; E, number of events; MR, measles rubella; N, the total number of subjects in each treatment group under the safety population; n, number of subjects reported AEs.

% = percentage of subjects; 95% CI = 95% confidence interval for the proportion of subjects.

The 95% CI estimated using the Clopper-Pearson exact method.

Subjects are counted only once within each preferred term and system organ class.

Percentages are based on the age subsets and treatments of Subjects in the specified treatment group under safety population.

AEs will be coded using the Medical Dictionary of Regulatory Activities (MedDRA version 24.1).

Overall, BE's TCV TYPHIBEV was found to be tolerable in age groups ranging from 6 months to 45 years, and no safety signals were identified.

### Immunogenicity findings

In this study, 400 children aged 9-12 months were randomly allocated 1:1 to receive either the TYPHIBEV^Ⓡ^ and MR vaccine or the MR vaccine only. An additional 132 children aged ≥6 months to <2 years in this age subset received only TYPHIBEV^Ⓡ^. Anti-measles IgG levels were assessed in the TYPHIBEV^Ⓡ^ + MR and MR only arms, and anti-Vi IgG levels were assessed in the TYPHIBEV^Ⓡ^ + MR and TYPHIBEV only arms. All immunogenicity assessments were conducted in the per-protocol population.

**Figure 2:**
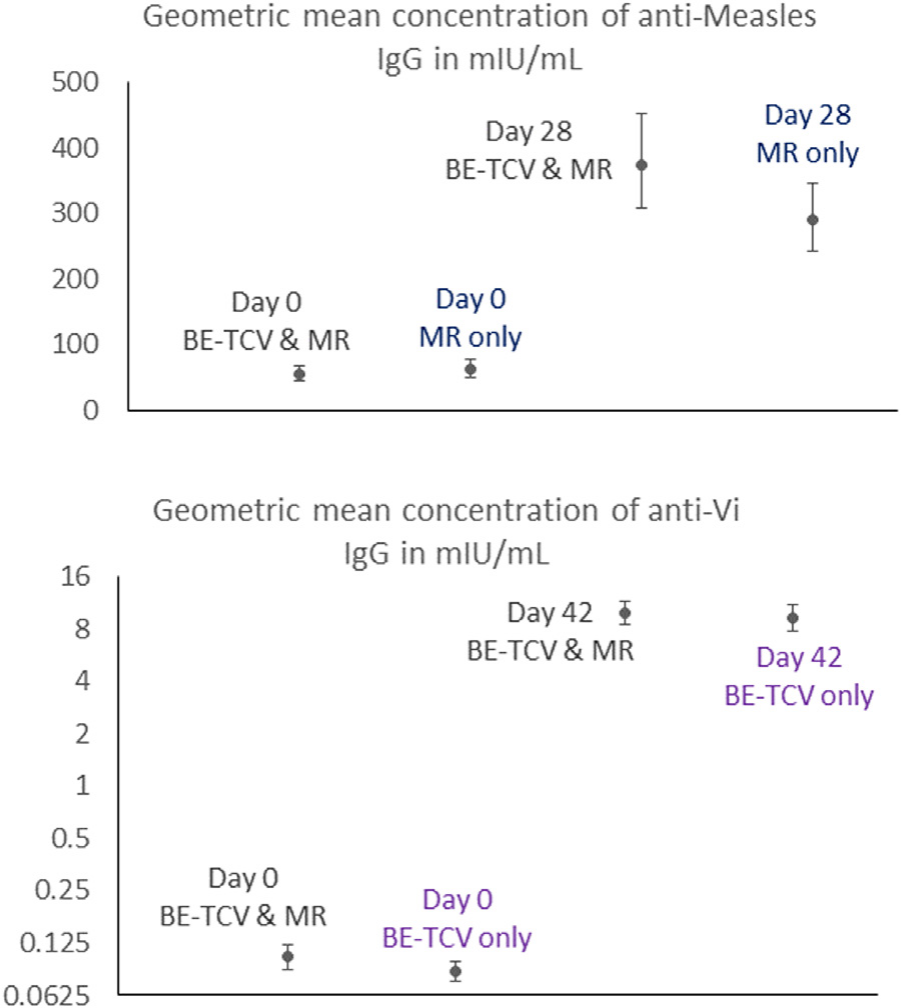
Geometric mean concentrations of anti-measles IgGs (upper panel) and anti-Vi IgGs (lower panel) after a single dose of TYPHIBEV in the phase IV study. Ig, immunoglobulin; MR, measles rubella; TCV, typhoid conjugate vaccine.

Anti-measles IgG antibody concentrations increased from baseline to day 28 in both arms, and GMCs were comparable between the TYPHIBEV^Ⓡ^ + MR arm (763.93 mIU/ml) and the MR only arm (506.96 mIU/ml). Similarly, anti-Vi IgG antibody concentrations increased substantially from baseline to day 42 in both relevant arms, reaching comparable levels in the TYPHIBEV^Ⓡ^ + MR arm (15.91 µg/ml) and TYPHIBEV^Ⓡ^ only arm (14.42 µg/ml). These data are visually represented in Figure 2 and demonstrate no evidence of immunogenic interference between the measles and typhoid components of the two vaccines when administered together.

The seroprotection rates for measles were similar between arms at day 28: 80.61% for TYPHIBEV^Ⓡ^ + MR versus 80.90% for MR only. The percentage of participants with ≥4-fold antibody increases was also comparable: 63.27% for TYPHIBEV^Ⓡ^ + MR vs 59.8% for MR only. For typhoid, seroprotection rates were nearly identical: 91.24% for TYPHIBEV^Ⓡ^ + MR versus 90.15% for TYPHIBEV^Ⓡ^ only. The percentage of subjects with ≥4-fold antibody increases was high in both arms: 96.39% for TYPHIBEV^Ⓡ^ + MR vs 96.97% for TYPHIBEV^Ⓡ^ only. All immunogenicity data are presented in a tabular form in Supplementary Tables 4-8.

Taken together, these descriptive data suggest that the co-administration of TYPHIBEV and MR vaccines elicit immune responses without any interference between the Vi and measles antigenic components. No safety signals were identified in the co-administration arm.

## Discussion

This study aimed to evaluate the safety and tolerability profile of the single intramuscular dose of BE's TYPHIBEV^Ⓡ^ in infants aged ≥6 months to adults aged 45 years and assess the potential immunogenic interference with measles-containing vaccines. MR/ Measles, Mumps, and Rubella vaccine (MMR) forms an important part of immunization programs across the world, and its first dose is usually given around the 9th month of life. It is important to assess the safety and immunogenicity of TYPHIBEV^Ⓡ^ co-administered with a measles-containing vaccine. In our study, we had three arms where we assessed the potential immunogenic interference of TYPHIBEV^Ⓡ^ and MR on each other. The study arms had participants receiving either TYPHIBEV^Ⓡ^ only, MR only, or both vaccines. Immunogenicity-wise, we show that the geometric mean concentration of IgG antibodies against measles (day 28) and the Vi antigen (day 42) increase from baseline after vaccinations. The elicited immune responses in the co-administration group were similar to the levels of participants who received either one of the two vaccines. Interestingly, the levels of anti-measles IgG antibodies were higher in the co-administration arm than in the MR only arm, although this difference was not statistically significant. This immunogenic non-interference finding is in line with other similar studies. Saluja *et al.* published their findings of a TCV co-administered with MMR and showed that the seropositivity levels for anti-measles, anti-mumps, and anti-rubella were similar in their MMR-only and MMR plus TCV co-administration arms [13]. Similarly, Sirima *et al.* showed that the anti-measles and anti-rubella antibody titers were similar in their TCV and MMR arm compared with their control arm [14]. Another study showed that TCV could be safely co-administered with the routine group A Meningococcal conjugate vaccine without interference [15].

Consistent with other co-administrations studies of typhoid conjugate vaccines [13,15], our findings also demonstrate the acceptable safety profile of TYPHIBEV^Ⓡ^ when co-administered with MR. The frequency of participants who had AEs after receiving TYPHIBEV^Ⓡ^ and MR was 16%, whereas it was 20.5% for those who received MR only. There were five unsolicited events in the TYPHIBEV^Ⓡ^ and MR in the form of diarrhea, vomiting, and a case of rash. There was one serious AE in the TYPHIBEV^Ⓡ^ and MR arm not related to either of the vaccines.

Before these studies, TYPHIBEV^Ⓡ^ was evaluated in a phase I study in healthy adult male volunteers aged 18 to 45 years, and the vaccine was found to have an acceptable safety profile while also being immunogenic after a single-dose administration. A phase II/III study was also conducted to evaluate the immunogenicity and safety of TYPHIBEV^Ⓡ^ conjugate vaccine compared with the currently licensed Typbar-TCV vaccine (Bharat Biotech). An additional phase III safety study was conducted exclusively to assess the continued safety of TYPHIBEV^Ⓡ^. These studies, when taken together, have demonstrated the overall safety of TYPHIBEV^Ⓡ^. Pain at the injection site was the most common reported AE in all these studies. Although few of the solicited events were medically attended, the severity was of all these events reported were mild or grade 1, except one with moderate severity. This event was unlikely to be related to the study vaccination(s). The large phase III safety study established that TYPHIBEV^Ⓡ^ was similar to the licensed comparator regarding AE rates. This phase IV study confirms and extends these findings. There was one serious AE in this study, although given the timing of the case, it was ruled to be unlikely to be related to the vaccine. Overall, BE-TCV has an acceptable safety and tolerability profile.

Despite the availability of polysaccharide conjugation technology for approximately 40 years, millions of people have continued to become infected and die from the disease. A recent commentary in the Lancet Global Health by Shakya and Pollard [16] discuses a systematic review and meta-analysis by Rabab Batool *et al.* highlighting the efficacy and safety of TCVs in preventing culture-confirmed *S.* Typhi infections [17]. Although high-income countries have successfully eliminated typhoid, the disease continues to disrupt the lives of children in settings with inadequate water quality and poor sanitation. The increasing prevalence of antimicrobial resistance and limited access to medical care in resource-poor and isolated communities further exacerbate the impact of typhoid fever. Moreover, climate change has been linked to a heightened risk of typhoid, as evidenced by the 2022 floods in Pakistan, where TCVs emerged as potent tools in alleviating the burden of the disease and combating the spread of drug-resistant *S.* Typhi [17]. TCVs, such as TYPHIBEV, are certainly going to be an important part of immunization campaigns in the Global South but may also turn out to be important in the Global North owing to changing climate patterns.

Although this study provides valuable data on the safety and co-administration of TYPHIBEV^Ⓡ^, it is important to acknowledge its limitations. As with most clinical trials, the sample size may not be sufficient to detect very rare AEs. Ongoing post-marketing surveillance and large-scale observational studies will be essential to monitor the long-term safety of TYPHIBEV^Ⓡ^ and identify any potential very rare AEs. Another limitation was the anti-measles immunogenicity assay used in this study. For the measurement of post-measles vaccination immune responses, the plaque reduction neutralization test is the preferred assay. In this study, we used an enzyme-linked immunosorbent assay–based anti-measles antibody measurement, which is often accepted as a surrogate for plaque reduction neutralization test titers. Finally, the immunogenic non-interference end point in this study was not formally powered for non-inferiority. The sample size was to be able to use descriptive statistics to compare the groups. In a *post hoc* analysis, the proportion of subjects seroprotected by anti-measles IgG antibodies after BE-TCV and MR was non-inferior to those seroprotected after MR only.

Through the various studies of TYPHIBEV^Ⓡ^, we have demonstrated an acceptable safety profile, and, with this study, we show a similar safety profile when co-administered with measles-containing vaccines. Although TYPHIBEV^Ⓡ^ has been shown to be robustly immunogenic in multiple studies, large efficacy studies are still needed. Given its WHO-prequalification status, large real-world studies of TYPHIBEV^Ⓡ^ could demonstrate its effectiveness, particularly, in regions with endemic typhoid. The ongoing Vellore Typhoid Vaccine Trial, an observer-blinded, cluster randomised trial may shed some light on the effectiveness of TYPHIBEV^Ⓡ^ (doi: 10.1186/s13063-023-07555-y).

In conclusion, in this phase IV study, we show an acceptable safety profile of TYPHIBEV^Ⓡ^ when co-administered with measles-containing vaccines, with no immunogenic interference. We also present additional data demonstrating an acceptable safety profile of TYPHIBEV^Ⓡ^ when administered to children as young as 6 months and adults as old as 45 years.

## Declarations of competing interest

ST, RVM, SRG, RRM, VV, PVS, CD, and NB are all employees of BE Limited and do not own any shares of the company. All other authors report no conflicts of interest.

## Appendix Supplementary materials

Image, application 1

## Footnotes

Supplementary material associated with this article can be found, in the online version, at doi:10.1016/j.ijregi.2025.100611.
