# Supplementary material for: Safety and immunogenicity of Biological E's typhoid conjugate vaccine TYPHIBEVⓇ concomitantly administered with measles rubella vaccine: a phase IV prospective, multicenter study
Source: IJID Reg. 2025 Feb 25;15:100611. doi: 10.1016/j.ijregi.2025.100611 (PMC11964491; doi:10.1016/j.ijregi.2025.100611)
Supplement: Supplementary file 1 [file mmc1.docx]

**Supplementary Table 1: Medical history and disease characteristics at baseline – ITT population of Phase III study**

| **SOC/PT, N1 (%)** [[1](#ref1)] | **≥6 months to <2 years (N=590)** | **≥2 years to <18 years (N=590)** | **≥18 years to <45 years (N=590)** | **Overall (N=1770)** |
| --- | --- | --- | --- | --- |
| Congenital, familial and genetic disorders | 0 (0.0%) | 1 (0.17%) | 0 (0.0%) | 1 (0.06%) |
| Keratosis follicular | 0 (0.0%) | 1 (0.17%) | 0 (0.0%) | 1 (0.06%) |
| Ear and labyrinth disorders | 1 (0.17%) | 0 (0.0%) | 0 (0.0%) | 1 (0.06%) |
| Ear pain | 1 (0.17%) | 0 (0.0%) | 0 (0.0%) | 1 (0.06%) |
| Otorrhea | 1 (0.17%) | 0 (0.0%) | 0 (0.0%) | 1 (0.06%) |
| Eye disorders | 0 (0.0%) | 1 (0.17%) | 0 (0.0%) | 1 (0.06%) |
| Lacrimation increased | 0 (0.0%) | 1 (0.17%) | 0 (0.0%) | 1 (0.06%) |
| Infections and infestations | 1 (0.17%) | 1 (0.17%) | 0 (0.0%) | 2 (0.11%) |
| Nasopharyngitis | 1 (0.17%) | 0 (0.0%) | 0 (0.0%) | 1 (0.06%) |
| Viral infection | 0 (0.0%) | 1 (0.17%) | 0 (0.0%) | 1 (0.06%) |
| Investigations | 1 (0.17%) | 0 (0.0%) | 0 (0.0%) | 1 (0.06%) |
| Parasite stool test positive | 1 (0.17%) | 0 (0.0%) | 0 (0.0%) | 1 (0.06%) |
| Respiratory, thoracic and mediastinal disorders | 1 (0.17%) | 1 (0.17%) | 0 (0.0%) | 2 (0.11%) |
| Cough | 1 (0.17%) | 1 (0.17%) | 0 (0.0%) | 2 (0.11%) |
| Oropharyngeal pain | 0 (0.0%) | 1 (0.17%) | 0 (0.0%) | 1 (0.06%) |
| Skin and subcutaneous tissue disorders | 1 (0.17%) | 1 (0.17%) | 0 (0.0%) | 2 (0.11%) |
| Dermatitis atopic | 0 (0.0%) | 1 (0.17%) | 0 (0.0%) | 1 (0.06%) |
| Skin hypopigmentation | 1 (0.17%) | 0 (0.0%) | 0 (0.0%) | 1 (0.06%) |
| Note**:** Percentages were calculated using column header group count as denominator.  N_1_: Subject Count, N: Sample Size. | | | | |

**Supplementary Table 2: Summary of Concomitant Medication – Safety Population (N=1252) of Phase IV study**

| **ATC Class  Preferred Term** | **Age Subset-I** | | | **Age Subset-II** | **Age Subset-III** |  |
| --- | --- | --- | --- | --- | --- | --- |
|  | **Subgroup-I MR + TYPHIBEV^®^  (N = 200)**  **n (%)** | **Subgroup-II MR (N = 200)**  **n (%)** | **Subgroup-III TYPHIBEV^®^ (N = 132)**  **n (%)** | **TYPHIBEV^®^ (N = 360)**  **n (%)** | **TYPHIBEV^®^ (N = 360)**  **n (%)** | **Total (N = 1252)**  **n (%)** |
| Number of Subjects with at Least One Medication Recorded | 7 (3.5) | 6 (3.0) | 4 (3.0) | 4 (1.1) | 7 (1.9) | 28 (2.2) |
| Anilides | 5 (2.5) | 5 (2.5) | 4 (3.0) | 4 (1.1) | 3 (0.8) | 21 (1.7) |
| Paracetamol | 5 (2.5) | 5 (2.5) | 4 (3.0) | 4 (1.1) | 3 (0.8) | 21 (1.7) |
| Antibiotics | 1 (0.5) | 0 (0.0) | 0 (0.0) | 0 (0.0) | 0 (0.0) | 1 (0.1) |
| Colistin Sulfate | 1 (0.5) | 0 (0.0) | 0 (0.0) | 0 (0.0) | 0 (0.0) | 1 (0.1) |
| Antidiarrheal Microorganisms | 3 (1.5) | 1 (0.5) | 0 (0.0) | 0 (0.0) | 0 (0.0) | 4 (0.3) |
| Bacillus Clausii | 1 (0.5) | 0 (0.0) | 0 (0.0) | 0 (0.0) | 0 (0.0) | 1 (0.1) |
| Bacillus Coagulans;bacillus  Mesentericus;clostridium  Butyricum;enterococcus Faecalis | 2 (1.0) | 1 (0.5) | 0 (0.0) | 0 (0.0) | 0 (0.0) | 3 (0.2) |
| Anti-inflammatory Preparations, Non-Steroids For Topical Use | 0 (0.0) | 0 (0.0) | 0 (0.0) | 0 (0.0) | 1 (0.3) | 1 (0.1) |
| Diclofenac Sodium | 0 (0.0) | 0 (0.0) | 0 (0.0) | 0 (0.0) | 1 (0.3) | 1 (0.1) |
| Imidazole And Triazole Derivatives | 0 (0.0) | 0 (0.0) | 0 (0.0) | 0 (0.0) | 1 (0.3) | 1 (0.1) |
| Clotrimazole | 0 (0.0) | 0 (0.0) | 0 (0.0) | 0 (0.0) | 1 (0.3) | 1 (0.1) |
| Imidazole Derivatives | 1 (0.5) | 0 (0.0) | 0 (0.0) | 0 (0.0) | 0 (0.0) | 1 (0.1) |
| Metronidazole | 1 (0.5) | 0 (0.0) | 0 (0.0) | 0 (0.0) | 0 (0.0) | 1 (0.1) |
| Leukotriene Receptor Antagonists | 0 | 0 (0.0) | 0 (0.0) | 0 (0.0) | 1 (0.3) | 1 (0.1) |
| Levocetirizine  Dihydrochloride; montelukast  Sodium | 0 (0.0) | 0 (0.0) | 0 (0.0) | 0 (0.0) | 1 (0.3) | 1 (0.1) |
| Macrolides | 0 (0.0) | 1 (0.5) | 0 (0.0) | 0 (0.0) | 0 (0.0) | 1 (0.1) |
| Azithromycin | 0 (0.0) | 1 (0.5) | 0 (0.0) | 0 (0.0) | 0 (0.0) | 1 (0.1) |
| Multivitamins, Plain | 1 (0.5) | 0 (0.0) | 0 (0.0) | 0 (0.0) | 0 (0.0) | 1 (0.1) |
| Vitamins Nos | 1 (0.5) | 0 (0.0) | 0 (0.0) | 0 (0.0) | 0 (0.0) | 1 (0.1) |
| Oral Rehydration Salt Formulations | 3 (1.5) | 1 (0.5) | 0 (0.0) | 0 (0.0) | 0 (0.0) | 4 (0.3) |
| Glucose;potassium  Chloride;sodium Chloride;sodium  Citrate | 3 (1.5) | 1 (0.5) | 0 (0.0) | 0 (0.0) | 0 (0.0) | 4 (0.3) |
| Other Agents For Local Oral Treatment | 1 (0.5) | 0 (0.0) | 0 (0.0) | 0 (0.0) | 0 (0.0) | 1 (0.1) |
| Benzalkonium Chloride;choline  Salicylate | 1 (0.5) | 0 (0.0) | 0 (0.0) | 0 (0.0) | 0 (0.0) | 1 (0.1) |
| Other Antiemetics | 1 (0.5) | 0 (0.0) | 0 (0.0) | 0 (0.0) | 0 (0.0) | 1 (0.1) |
| Promethazine | 1 (0.5) | 0 (0.0) | 0 (0.0) | 0 (0.0) | 0 (0.0) | 1 (0.1) |
| Other Antipruritics | 1 (0.5) | 1 (0.5) | 0 (0.0) | 0 (0.0) | 3 (0.8) | 5 (0.4) |
| Calamine | 1 (0.5) | 1 (0.5) | 0 (0.0) | 0 (0.0) | 3 (0.8) | 5 (0.4) |
| Other Emollients And Protectives | 1 (0.5) | 0 (0.0) | 0 (0.0) | 0 (0.0) | 0 (0.0) | 1 (0.1) |
| Calamine;cetrimide;  dimeticone;zinc Oxide | 1 (0.5) | 0 (0.0) | 0 (0.0) | 0 (0.0) | 0 (0.0) | 1 (0.1) |
| Other Nasal Preparations | 2 (1.0) | 1 (0.5) | 0 (0.0) | 0 (0.0) | 0 (0.0) | 3 (0.2) |
| Sodium Chloride | 2 (1.0) | 1 (0.5) | 0 (0.0) | 0 (0.0) | 0 (0.0) | 3 (0.2) |
| Other Plain Vitamin Preparations | 1 (0.5) | 0 (0.0) | 0 (0.0) | 0 (0.0) | 0 (0.0) | 1 (0.1) |
| Vitamin B Nos | 1 (0.5) | 0 (0.0) | 0 (0.0) | 0 (0.0) | 0 (0.0) | 1 (0.1) |
| Penicillins With Extended Spectrum | 0 (0.0) | 0 (0.0) | 0 (0.0) | 0 (0.0) | 1 (0.3) | 1 (0.1) |
| Ambroxol  Hydrochloride;amoxicillin  Trihydrate | 0 (0.0) | 0 (0.0) | 0 (0.0) | 0 (0.0) | 1 (0.3) | 1 (0.1) |
| Phenothiazine Derivatives | 1 (0.5) | 1 (0.5) | 0 (0.0) | 0 (0.0) | 0 (0.0) | 2 (0.2) |
| Promethazine | 1 (0.5) | 1 (0.5) | 0 (0.0) | 0 (0.0) | 0 (0.0) | 2 (0.2) |
| Piperazine Derivatives | 0 (0.0) | 1 (0.5) | 0 (0.0) | 0 (0.0) | 4 (1.1) | 5 (0.4) |
| Cetirizine | 0 (0.0) | 0 (0.0) | 0 (0.0) | 0 (0.0) | 1 (0.3) | 1 (0.1) |
| Cetirizine Hydrochloride | 0 (0.0) | 1 (0.5) | 0 (0.0) | 0 (0.0) | 0 (0.0) | 1 (0.1) |
| Levocetirizine | 0 (0.0) | 0 (0.0) | 0 (0.0) | 0 (0.0) | 3 (0.8) | 3 (0.2) |
| Serotonin (5ht3) Antagonists | 1 (0.5) | 0 (0.0) | 0 (0.0) | 0 (0.0) | 0 (0.0) | 1 (0.1) |
| Ondansetron Hydrochloride | 1 (0.5) | 0 (0.0) | 0 (0.0) | 0 (0.0) | 0 (0.0) | 1 (0.1) |
| Solutions Affecting The Electrolyte Balance | 1 (0.5) | 0 (0.0) | 0 (0.0) | 0 (0.0) | 0 (0.0) | 1 (0.1) |
| Calcium Chloride  Dihydrate; Potassium  Chloride; Sodium Chloride;  Sodium Lactate | 1 (0.5) | 0 (0.0) | 0 (0.0) | 0 (0.0) | 0 (0.0) | 1 (0.1) |
| Third-Generation Cephalosporins | 2 (1.0) | 0 (0.0) | 0 (0.0) | 0 (0.0) | 0 (0.0) | 2 (0.2) |
| Cefixime | 1 (0.5) | 0 (0.0) | 0 (0.0) | 0 (0.0) | 0 (0.0) | 1 (0.1) |
| Ceftriaxone | 1 (0.5) | 0 (0.0) | 0 (0.0) | 0 (0.0) | 0 (0.0) | 1 (0.1) |
| Triazole Derivatives | 0 (0.0) | 0 (0.0) | 0 (0.0) | 0 (0.0) | 1 (0.3) | 1 (0.1) |
| Fluconazole | 0 (0.0) | 0 (0.0) | 0 (0.0) | 0 (0.0) | 1 (0.3) | 1 (0.1) |
| Various Alimentary Tract And Metabolism Products | 3 (1.5) | 1 (0.5) | 0 (0.0) | 0 (0.0) | 0 (0.0) | 4 (0.3) |
| Zinc | 3 (1.5) | 1 (0.5) | 0 (0.0) | 0 (0.0) | 0 (0.0) | 4 (0.3) |
| Vitamin D And Analogues | 2 (1.0) | 0 (0.0) | 0 (0.0) | 0 (0.0) | 0 (0.0) | 2 (0.2) |
| Colecalciferol | 2 (1.0) | 0 (0.0) | 0 (0.0) | 0 (0.0) | 0 (0.0) | 2 (0.2) |

**Supplementary Table 3: Prior vaccination history – ITT population (N=1252) of Phase IV study**

| **Vaccine name** | **Age Subset-I** | | | **Age Subset-II**  **TYPHIBEV^®^ (N = 360)**  **n (%)** | **Age Subset-III**  **TYPHIBEV^®^ (N = 360)**  **n (%)** | **Total (N = 1252)**  **n (%)** |
| --- | --- | --- | --- | --- | --- | --- |
|  | **Subgroup-I MR + TYPHIBEV^®^  (N = 200)**  **n (%)** | **Subgroup-II MR (N = 200)**  **n (%)** | **Subgroup-III TYPHIBEV^®^ (N = 132)**  **n (%)** |  |  |  |
| BCG | 198 (99.0) | 200 (100) | 132 (100) | 360 (100) | 360 (100) | 1250 (99.8) |
| DTP | 200 (100) | 200 (100) | 132 (100) | 350 (97.2) | 355 (98.6) | 1237 (98.8) |
| HEP-A | 3 (1.5) | 5 (2.5) | 4 (3.0) | 8 (2.2) | 86 (23.9) | 106 (8.5) |
| HEPATITIS-B | 200 (100) | 198 (99.0) | 132 (100) | 348 (96.7) | 271 (75.3) | 1149 (91.8) |
| HIB | 199 (99.5) | 196 (98.0) | 132 (100) | 293 (81.4) | 204 (56.7) | 1024 (81.8) |
| COVID 19 | 0 (0.0) | 0 (0.0) | 0 (0.0) | 0 (0.0) | 17 (4.7) | 17 (1.4) |
| FLU | 0 (0.0) | 0 (0.0) | 0 (0.0) | 3 (0.8) | 0 (0.0) | 3 (0.2) |
| ROTA | 21 (43.5) | 73 (36.5) | 33 (25.0) | 0 (0.0) | 0 (0.0) | 127 (10.1) |
| VITAMIN A | 0 (0.0) | 2 (1.0) | 0 (0.0) | 0 (0.0) | 0 (0.0) | 2 (0.2) |
| PCV | 172 (86.0) | 174 (87.0) | 108 (81.8) | 202 (56.1) | 72 (20.0) | 728 (58.1) |
| POLIO | 200 (100) | 200 (100) | 132 (100) | 360 (100) | 360 (100) | 1252 (100) |
| VARICELLA | 3 (1.5) | 4 (2.0) | 4 (3.0) | 8 (2.2) | 82 (22.8) | 101 (8.1) |

**Supplementary Table 4: Summary of Geometric mean concentrations of anti-Measles IgG antibodies – Per Protocol Population (N=395)**

| **Visit / Day** | **Statistics** | **Subgroup-1**  **BE-TCV + MR (N =196)** | **Subgroup-2**  **MR only (N =199)** |
| --- | --- | --- | --- |
| Baseline (Day 0) | n | 196 | 199 |
|  | Mean | 301.363 | 437.568 |
|  | Geometric Mean | 55.840 | 62.560 |
|  | 95% CI of Geometric Mean | (45.733, 68.180) | (49.765, 78.645) |
|  | (Min, Max) | (25.00, 4712.41) | (25.00, 4870.20) |
|  | Median | 25.00 | 25.00 |
|  | (Q1, Q3) | (25.00, 88.14) | (25.00, 94.49) |
| Day 28 | n | 196 | 199 |
|  | Mean | 763.926 | 506.957 |
|  | Geometric Mean | 372.541 | 288.989 |
|  | 95% CI of Geometric Mean | (307.338, 451.577) | (242.390, 344.548) |
|  | (Min, Max) | (25.00, 15405.2) | (25.00, 2726.11) |
|  | Median | 510.95 | 424.02 |
|  | (Q1, Q3) | (195.89, 953.05) | (151.01, 801.92) |

**Supplementary Table 5: Summary of Geometric mean concentrations of anti-Vi IgG antibodies – Per Protocol Population (N=328)**

| **Visit / Day** | **Statistics** | **Subgroup-1**  **BE-TCV + MR (N =194)** | **Subgroup-3**  **BE-TCV only only (N =132)** |
| --- | --- | --- | --- |
| Baseline (Day 0) | n | 194 | 132 |
|  | Mean | 0.928 | 0.281 |
|  | Geometric Mean | 0.104 | 0.086 |
|  | 95% CI of Geometric Mean | (0.088, 0.122) | (0.075, 0.098) |
|  | (Min, Max) | (0.07, 56.41) | (0.07, 21.24) |
|  | Median | 0.07 | 0.07 |
|  | (Q1, Q3) | (0.07, 0.07) | (0.07, 0.07) |
| Day 42 | n | 194 | 132 |
|  | Mean | 15.913 | 14.423 |
|  | Geometric Mean | 9.973 | 9.249 |
|  | 95% CI of Geometric Mean | (8.574, 11.599) | (7.782, 10.993) |
|  | (Min, Max) | (0.52, 191.59) | (1.01, 85.58) |
|  | Median | 11.96 | 10.75 |
|  | (Q1, Q3) | (5.74, 18.66) | (5.08, 17.11) |

**Supplementary Table 6: Proportion of subjects seroprotected of anti-Measles IgG antibodies – Per Protocol Population (N=395)**

| **Visit** | **Subgroup-1**  **BE-TCV + MR (N = 196)**  **n (%) [95% CI]** | **Subgroup-2**  **MR only (N =199)**  **n (%) [95% CI]** | |
| --- | --- | --- | --- |
| Day 0 | 46 (23.47) [17.72, 30.04] | | 46 (23.12) [17.45, 29.60] |
| Day 28 | 158 (80.61) [74.37, 85.90] | | 161 (80.90) [74.75, 86.12] |

**Supplementary Table 7: Proportion of subjects seroprotected of anti-Vi IgG antibodies – Per Protocol Population (N=328)**

| **Visit** | **Subgroup-1**  **BE-TCV + MR (N = 196)**  **n (%) [95% CI]** | **Subgroup-3**  **BE-TCV only (N =132)**  **n (%) [95% CI]** | |
| --- | --- | --- | --- |
| Day 0 | 6 (3.09) [1.14, 6.61] | | 1 (0.76) [0.00, 4.15] |
| Day 28 | 177 (91.24) [86.34, 94.81] | | 119 (90.15) [83.75, 94.65] |

**Supplementary Table 8: Proportion of subjects achieving ≥4-fold rise and GMFR from baseline of anti-Measles IgG antibodies – Per Protocol Population (N=395)**

| **Parameter** | **Subgroup-1**  **BE-TCV + MR (N = 196)**  **n (%) [95% CI]** | **Subgroup-2**  **MR only (N =199)**  **n (%) [95% CI]** | |
| --- | --- | --- | --- |
| ≥4-Fold Increase | 124 (63.27) [56.10, 70.02] | | 119 (59.80) [52.63, 66.67] |
| GMFR | 6.672 | | 4.619 |

**Supplementary table of study sites**

| **Site Code** | **Principal Investigator** | **Sub-Investigator** | **Site Address** |
| --- | --- | --- | --- |
| A | Dr. B.S Chakravarthy | Dr. Y. Gnana Sundhara Raju | King George Hospital (KGH), Near Collectorate Junction, Maharanipeta, Visakhapatnam – 530002  Andhra Pradesh, India. |
| C | Dr. N. Pradeep | Dr. Prashanth | Cheluvamba Hospital, Irwin Rd, Devraj Mohalla, Mysuru – 570001, Karnataka, India |
| D | Dr. Manish Narang | Dr. Shiva Narang | GTB Hospital, Tahirpur Rd, GTB Enclave, Dilshad, Garden, Delhi - 110095, India |
| E | Dr. Gaurav Sharma | Dr. Dilip Shah | Vijay Vallabh Hospital, Tirupati Nagar Rd, Beside Banjara Hotel, Phase 1, Tirupati Nagar, Virar West,  Mumbai - 401303, Maharashtra, India |
| F | Dr. Chandra Prakash Suthar | Dr. Sunil Kumar | Dana Shivam Heart & Superspeciality Hospital, Plot No:2, Opp. Times Square, Sector 2, Vijay Bari, Vidyadhar Nagar, Jaipur - 302023, Rajasthan, India |
| G | Dr. Satish Chandran | Dr.Roshni.K.S | Elite Mission Hospital, Koorkenchery Rd, Koorkenchery, Thrissur - 680007, Kerala, India |
| I | Dr. N. S. Mahantshetti | Dr. Sonali Bijjargi | KLES Dr. Prabhakar Kore Hospital & Medical Research Centre J N Medical College,Nehru Nagar,  Belagavi – 590010, Karnataka, India |
| J | Dr. Veer Bahadur Singh | Dr. Jayprakashnarayan | JLN Medical College,  Ajmer - 305001, Rajasthan, India |
| L | Dr. M. Alexander | Dr. Thirugnanasambandan | Chettinad Hospital and Research Institute, SH 49A, Kelambakkam, Chennai - 603103, Tamil Nadu, India |
| M | Dr. Anil Kumar Pandey | Dr. Sankalp | ESIC Medical College & Hospital, Room No. 440, 4th Floor, NH-3 behind BK Hospital New Industrial Town,  Faridabad - 121001, Haryana, India |
| N | Dr. Nishant Kumar Bansal | Dr. Sandeep Jain | Tagore hospital and research institute, Tagore lane, Sector-7, Shipra Path, Madhyam Marg, Manasarovar, Jaipur - 30202, Rajasthan, India |

Note: Initially 14 sites were activated but enrolment occurred only at 11 of these sites mentioned in the table.
